# Use of prescription medication prior to suicide in Norway

**DOI:** 10.1186/s12913-019-4009-1

**Published:** 2019-04-04

**Authors:** Anne Reneflot, Silje L. Kaspersen, Lars Johan Hauge, Jorid Kalseth

**Affiliations:** 1 Mental and Physical Health, Norwegian Institute of Public Health, PO Box 222, 0213 Skøyen, Oslo Norway; 2 SINTEF Digital, Department of Health, The Norwegian University of Science and Technology, Trondheim, Norway; 3 Department of Public Health and Nursing, The Norwegian University of Science and Technology, Trondheim, Norway

**Keywords:** Prescription medications, Psychotropic medication, Suicide, Population-based

## Abstract

**Background:**

The use of psychotropic medications in relation to mental disorders is considered central to preventing suicide. However, few studies have addressed prescription patterns at different time points within the last year prior to suicide and compared these with those of the general population.

**Methods:**

We use data covering the period from 2010 to 2011 from the Norwegian Cause of Death Registry and the Norwegian Prescription Database to examine dispensing patterns of prescription medication within 12 months and within 30 days of suicide. Our data includes all registered suicides in Norway among individuals aged 15 years and older in 2011 (*n* = 594), 434 men and 160 women. Dispensing of prescription medication in the general population (n ≈ 4 million) are used for comparison.

**Results:**

Dispensing of any prescription medication were high and varied from 95.6% for females and 83.2% for males within 12 months of suicide, to 64.4% for females and 47.2% for males within 30 days of suicide, respectively. The percentages with dispensed prescription medication increased with age. A similar sex and age pattern was observed for the dispensing of psychotropic medications. Within the last 30 days, close to one in two were dispensed psychotropic medications. The dispensing of antidepressants, hypnotics and sedatives was more common than the dispensing of other categories of psychotropics. The percentages with dispensed prescription medication among the population controls were considerably lower, in particular the dispensing of psychotropics.

**Conclusion:**

Dispensing of prescription medications, including psychotropic medications, is common prior to suicide. The percentage with dispensed prescription medication increases with age and are higher for females than for males.

## Background

Suicide is a significant public health problem and continues to be among the primary causes of premature death worldwide, particularly among men and young individuals [1, 2]. Given the high toll taken by suicidal behaviour, efforts to identify targeted and effective suicide prevention strategies remain pressing [2, 3].

Suicidal behaviour is a complex phenomenon, often involving mental disorders, psychosocial problems, as well as alcohol and substance abuse [4]. Individuals experiencing such problems are often in need of long-lasting and coordinated measures from both the healthcare and the welfare services [3, 5]. This need may include the prescription of medications.

Several studies report that healthcare use in the year and months prior to suicide is common [6–9]. While approximately two out of 10 have been in contact with mental healthcare within a month of suicide, more than four out of 10 have consulted the primary healthcare services [6, 7]. Females have higher contact rates than males, and contact increases with age. So far, studies examining prescription of medications prior to suicide are few, and in particular studies including population controls. To gain better knowledge of the help and treatment offered to those who die by suicide, information about their healthcare service use should be complemented with information regarding use of prescribed medications.

The use of psychotropic medications in relation to mental disorders is considered central to preventing suicide [2, 10]. The introduction of a new and less toxic generation of antidepressants in the 1990s, the selective serotonin re-uptake inhibitors (SSRIs), is often argued to be associated with reduced suicide rates. However, there is also a growing concern that psychotropic medications in some instances can increase suicidality [10–12]. This concern is not limited to psychotropic medications, but involves a wide range of other medications such as smoking cessation and antiepileptic drugs [13–15]. So far, studies summarizing the suicide risk associated with the latter medications have reported inconclusive results [15]. Bearing this concern in mind further underlines the importance of mapping prescription patterns in the time prior to suicide [16].

Previous studies find that being prescribed medication, in particular psychotropic medication, is common in the time prior to suicide. For example, based on information from medical records in the period 1970–1995, a Swedish study examined the prescription of psychotropics in 59 suicides occurring between 1985 and 1995 [17]. Three months prior to suicide, 61% of males and 77% of females were prescribed any kind of drugs, whereas 37% of males and 31% of females were prescribed psychotropics, respectively [17]. A study from Northern Ireland based on coroner data of 1371 suicides occurring between 2005 and 2011 found that the majority of those who died by suicide were prescribed at least one medication prior to the suicide (65.1%) [18]. About half were prescribed psychotropic medications (51.7%) [18]. In an English study based on data from primary care of 2384 suicides occurring between 2002 and 2011, 38.5% were prescribed antidepressants in the year preceding suicide [19]. Moreover, the existing literature report a sex and age pattern. The percentage with a prescription are reported to increase with age and to be higher among females than among males [18]. The most frequently prescribed groups of medications were antidepressants and hypnotic/anxiolytic medications [17, 18].

A weakness of the existing literature is that very little is known about the prescription patterns at various points in time in the period leading up to suicide, and how such patterns may vary across sex and age. Moreover, in evaluating prescription patterns prior to suicide, it is important to include control groups for comparison. Most studies so far have not included control groups. The Swedish and English studies referred above, make two exceptions [17, 19]. Henriksson and colleagues utilised a case-control design where comparisons were made from the start of the observation period in 1970 until the date of suicide occurring between 1985 and 1995. In this period, those who died by suicide were 1.5 times more likely to be prescribed any drug, and more than twice as likely to be prescribed psychotropic drugs [17]. In their study, Windfuhr and colleagues reported that in the year preceding suicide, those who died by suicide were almost four times more likely to be prescribed antidepressants than the living controls [19]. However, similar analyses closer in time to the suicide such as the last month is still lacking. From a prevention perspective, information from this last period is vital.

### Aims of the study

In this paper we use Norwegian registry data covering all suicides in Norway in 2011 in order to examine the dispensing of prescription medications prior to suicide. Information about the date and cause of death is linked with information on the date and type of dispensed prescribed medications. We examine the dispensing of any prescription medication and the dispensing of psychotropics within 12 months and within 30 days of suicide according to sex and age. Data on dispensed prescription medications in the general population during the corresponding period is used for comparison.

## Methods

### Data and study population

This study is based on data covering the period from 2010 to 2011 from the Norwegian Cause of Death Registry [20] and the Norwegian Prescription Database [21]. By means of the unique personal identification number assigned to all Norwegian residents, it is possible to construct individual record linkages between the two data sources. The data include all registered suicides in Norway among individuals aged 15 years and older in 2011 (*n* = 594), 434 men and 160 women. This constitute 98.8% of all suicides in Norway during the respective year. Table 1 presents the distribution of suicides by sex and age. Age was categorized as 15–29, 30–44, 45–64, and 65 years and older.

**Table 1:** Suicides and control group (mid-year population) by age and sex in 2011

Age	Men				Women				Total			
	Control		Suicide		Control		Suicide		Control		Suicide	
	%	n	%	n	%	n	%	n	%	n	%	n
15–29	24,5	495,097	20,0	87	23,2	472,463,5	19,4	31	23,8	967,560,5	19,9	118
30–44	26,4	534,492,5	29,3	127	25,0	509,173,5	26,3	42	25,7	1,043,666	28,5	169
45–64	31,9	645,998	34,6	150	30,4	620,214,5	40,6	65	31,2	1,266,212,5	36,2	215
65+	17,2	348,534,5	16,1	70	21,4	437,091	13,8	22	19,3	785,625,5	15,5	92
Total	100	2,024,122	100	434	100	2,038,942,5	100	160	100	4,063,064,5	100	594

Information about the date and cause of death, age and sex were obtained from the Norwegian Cause of Death Registry. Suicide was coded according to the ICD-10 codes X60-X84, and Y87.0 [22]. From the Norwegian Prescription Database we obtained information on dispensing date and type of drug (purchased from pharmacies), coded according to the Anatomical Therapeutic Chemical (ATC) classification system [23]. For comparison purposes, we also obtained data from the Norwegian Prescription Database on dispensed prescription medications in the general population in 2011. Medication used in hospitals and nursing homes is not recorded in the registry and thus not included.

### Analysis

We calculated the percentages of individuals with any dispensed prescription, of antidepressants (N06A), psycholeptics ((antipsychotics (N05A), anxiolytics (N05B) and hypnotics and sedatives (N05C)), and any of the former psychotropic medications (N06A, N05A, N05B and N05C), within 12 months and within 30 days of suicide. The 12 months percentages in the control group were calculated using dispensing figures in 2011 in the general population and the mid-year population. To calculate the 30 days percentages we used monthly dispensing figures in 2011 to calculate the average monthly dispensing percentages this year.

As controls, we included the dispensing of prescription medications in the general population in 2011. Percentages of dispensed prescription medications during 30 days in the general population were calculated as the average over all calendar months in 2011. Results for cells with less than five observations are not reported.

We tested whether the percentages with dispensed prescription medications were higher among men and women who died by suicide than among the controls using the z-test. Significant differences are reported on a 5% level (z ≥ 1.96).

### Ethical approval

The Regional Committee for Medical and Health Research Ethics granted approval for the research project (approval number 2012/852), and both registry owners consented to their data being utilized.

## Results

Table 1 shows the distribution of suicides and the general population by age and sex. In 2011 there were 594 suicides among Norwegians aged 15 years and older, 434 males and 160 females.

### Dispensing of any prescription medication

Table 2 shows the percentages with any dispensed prescription medication within 12 months and within 30 days of suicide by sex and age. On average, 95.6% of females and 83.2% of males were prescribed any medication within 12 months of suicide. Corresponding percentages within 30 days of suicide were 64.4% for females, and 47.2% for males, respectively. At both time points, the percentages with dispensed prescription medication increased with age for males, while for female this pattern was only evident within 30 days of the suicide. Compared with the general population, the percentages with dispensed prescription medication in the suicide group were higher for both sexes and across all age groups. However, among the oldest women, the observed difference were not significant. In the general population, the percentages with dispensed prescription medication varied from 82.8% for females and 66.1% for males within the last 12 months, to 34.4% for females and 23.3% for males within the last 30 days.

**Table 2:** Proportion (%) of the suicide population and the general population (control) that were dispensed any medication within 12 months and within 30 days of suicide by gender and age

	12 months					30 days				
	Control		Suicide			Control		Suicide		
Males	%	n	%	n	z-scores^a^	%	n	%	n	z-scores^a^
15–29	48.7	241,150	66.7	58	**3.36**	9.8	48,688	21.8	19	**3.76**
30–44	57.6	307,668	79.5	101	**4.99**	15.1	80,871	39.4	50	**7.65**
45–64	73.0	471,363	89.3	134	**4.50**	27.3	176,226	54	81	**7.34**
65+	91.3	318,169	97.1	68	1.72	47.7	166,395	78.6	55	**5.18**
Total	66.1	1,338,350	83.2	361	**7.53**	23.3	472,180	47.2	205	**11.78**
Females	%	n	%	n	z-scores^a^	%	n	%	n	z-scores^a^
15–29	79.1	373,900	96.8	30	**2.42**	25.1	118,770	45.2	14	**2.58**
30–44	77.6	395,188	92.9	39	**2.38**	25.1	127,687	66.7	28	**6.22**
45–64	83.9	520,022	95.4	62	**2.52**	35.9	222,390	70.8	46	**5.87**
65+	91.4	399,688	100	22	1.44	53.5	233,690	68.2	15	1.38
Total	82.8	1,688,798	95.6	153	**4.29**	34.4	702,537	64.4	103	**7.99**

^a^Significant z-scores on a 5% level is presented in bold

### Dispensing of psychotropic medications

Table 3 shows the percentages that were dispensed psychotropic medication within 12 months and within 30 days of suicide. Within the last 12 months, 74.4% of females and 62.9% of males were dispensed psychotropic medication. The corresponding percentages within 30 days of suicide were 50.6% for females and 36.2% for males, respectively. For males the percentages increased with age. This pattern was not as distinct for females. For both sexes and across all age groups, the percentages that were dispensed psychotropic medication among the population controls were considerably lower. In the general population, the percentages varied from 23.2% for females and 13.4% for males in the last 12 months, to 7.7% for females and 4.3% for males in the last 30 days.

**Table 3:** Proportion (%) of the suicide population and the general population (control) that were dispensed any psychotropics within 12 months and within 30 days of suicide by gender and age

	12 months					30 days				
	Control		Suicide			Control		Suicide		
Males	%	n	%	n	z-scores^a^	%	n	%	n	z-scores^a^
15–29	5.2	26,151	36.8	32	**13.28**	1.3	6629	14.9	13	**11.20**
30–44	10.3	55,072	60.6	77	**18.65**	3.2	17,138	28.3	26	**16.07**
45–64	15.8	101,912	72.7	109	**19.11**	5.3	34,169	44	66	**21.16**
65+	25.4	89,020	78.6	55	**10.23**	8.4	29,164	60	42	**15.56**
Total	13.4	272,155	62.9	273	**30.27**	4.3	87,100	36.2	157	**32.76**
Females	%	n	%	n	z-scores^a^	%	n	%	n	z-scores^a^
15–29	7.8	37,113	54.8	17	**9.76**	1.8	8854	29	9	**11.39**
30–44	15.2	77,395	71.4	30	**10.14**	4.6	23,146	52.4	22	**14.79**
45–64	27.3	169,323	83.1	54	**10.10**	9.3	57,508	61.5	40	**14.49**
65+	43.6	190,557	81.8	18	**3.61**	15.6	68,825	45.5	10	**3.86**
Total	23.2	474,388	74.4	119	**15.34**	7.7	158,333	50.6	81	**20.35**

^a^Significant z-scores on a 5% level is presented in bold

### Dispensing of psycholeptics

Tables 4, 5 and 6 shows the percentages that were dispensed antipsychotic, anxiolytic, and hypnotics and sedatives within 12 months and within 30 days of suicide. In the last year prior to suicide, 38.1% of females and 21.9% of males were dispensed antipsychotic medications. The corresponding percentages within 30 days of suicide were 16.3% for females and 9% for males, respectively. For anxiolytics, the percentages within 12 months of suicide were 42.5% for females and 34.6% males. Within the last 30 days before suicide, the percentages were 19.4% for females and 17.7% for males, respectively. Finally, the percentages that were dispensed hypnotics and sedatives in the last 12 months prior to suicide were 50% for females and 42.2% for males. The corresponding percentages 30 days prior to suicide were 28.1% for females and 21.2% for males, respectively.

**Table 4:** Proportion (%) of the suicide population and the general population (control) that were dispensed antipsychotics within 12 months and within 30 days of suicide by gender and age

	12 months					30 days				
	Control		Suicide			Control		Suicide		
Males	%	n	%	n	z-scores^a^	%	n	%	n	z-scores^a^
15–29	1.2	6009	14.9	13	**11.74**	0.3	1674	5.7	5	**9.21**
30–44	2.2	11,884	25.2	32	**17.67**	0.7	3866	9.4	12	**11.76**
45–64	2.7	17,442	25.3	38	**17.08**	0.9	5956	10.7	16	**12.71**
65+	2.9	10,219	17.1	12	**7.08**	0.9	3163	8.6	6	**6.82**
Total	2.2	45,554	21.9	95	**27.98**	0.7	14,659	9	39	**20.74**
Females	%	n	%	n	z-scores^a^	%	n	%	n	z-scores^a^
15–29	1.2	5845	22.6	7	**10.94**	0.3	1454	–	–	**–**
30–44	2.3	11,528	45.2	19	**18.55**	0.7	3498	19	8	**14.22**
45–64	3.4	21,327	41.5	28	**16.95**	1.2	7386	18.5	12	**12.81**
65+	4.4	19,259	36.4	8	**7.32**	1.4	6223	–	–	**–**
Total	2.8	57,959	38.1	91	**27.07**	0.9	18,561	16.3	26	**20.63**

^a^Significant z-scores on a 5% level is presented in bold

**Table 5:** Proportion (%) of the suicide population and the general population (control) that were dispensed anxiolytics within 12 months and within 30 days of suicide by gender and age

	12 months					30 days				
	Control		Suicide			Control		Suicide		
Males	%	n	%	n	z-scores^a^	%	n	%	n	z-scores^a^
15–29	1.4	7393	14.9	13	**10.72**	0.3	1590	5.7	5	**9.21**
30–44	3.5	18,785	35.4	45	**19.56**	1.1	5595	12.6	16	**12.43**
45–64	5.8	37,723	38.7	58	**17.24**	1.9	12,406	22.7	34	**18.66**
65+	8.9	31,080	48.6	34	**11.67**	2.4	8391	31.4	22	**15.85**
Total	4.6	94,981	34.6	150	**29.83**	1.3	27,982	17.7	77	**30.16**
Females	%	n	%	n	z-scores^a^	%	n	%	n	z-scores^a^
15–29	2.1	10,332	29	9	**10.45**	0.3	1748	–	–	**–**
30–44	5.1	26,092	38.1	16	**9.72**	1.3	6636	14.3	6	**7.44**
45–64	10.3	63,587	49.2	32	**10.32**	3.2	19,736	26.2	17	**10.54**
65+	17.3	75,384	50	11	**4.05**	5.1	22,307	–	–	**–**
Total	8.6	175,395	42.5	68	**15.29**	2.4	50,427	19.4	31	**14.05**

^a^Significant z-scores on a 5% level is presented in bold

**Table 6:** Proportion (%) of the suicide population and the general population (control) that were dispensed hypnotics and sedatives within 12 months and within 30 days of suicide by gender and age

	12 months					30 days				
	Control		Suicide			Control		Suicide		
Males	%	n	%	n	z-scores^a^	%	n	%	n	z-scores^a^
15–29	2.1	10,692	14.9	13	**8.33**	0.4	2104	5.7	5	**7.83**
30–44	4.3	23,132	32.3	41	**15.55**	1.1	5934	16.5	21	**16.64**
45–64	7.8	50,038	57.3	86	**22.61**	2.2	14,096	26.7	40	**20.46**
65+	16.1	56,098	61.4	43	**10.31**	4.6	15,847	37.1	26	**12.98**
Total	6.9	139,960	42.2	183	**29.01**	1.8	37,981	21.2	92	**30.40**
Females	%	n	%	n	z-scores^a^	%	n	%	n	z-scores^a^
15–29	2.8	13,640	25.8	8	**7.76**	0.5	2571	–	–	**–**
30–44	6.2	31,631	42.9	18	**9.86**	1.6	8146	21.4	9	**10.23**
45–64	15.0	92,732	60	39	**10.16**	4.2	26,203	36.9	24	**13.14**
65+	29.2	127,649	68.2	15	**4.02**	8.8	38,327	40.9	9	**5.31**
Total	13	265,652	50	80	**13.92**	3.6	75,247	28.1	45	**16.64**

^a^Significant z-scores on a 5% level is presented in bold

We observed an age gradient for anxiolytics, and hypnotics and sedatives. The age patterns for antipsychotics were somewhat different. The highest percentages were found in the age groups 30–44 years and 45–64 years.

The percentages that were dispensed psycholeptics among the population controls were strikingly lower. For antipsychotics, the percentages in the general population varied from 2.8% for females and 2.2% for males in the last 12 months, to 0.9% for females and 0.7% for males in the last 30 days. The percentages that were dispensed either anxiolytic medications, or hypnotics and sedatives within the last 12 months were 8.6 and 13% for females and 4.6 and 6.9% for males, respectively. The corresponding percentages for the last 30 days were 2.4 and 3.6% for females and 1.3 and 1.8% for males, respectively. Finally, it is worth noting that the age pattern for dispensing of antipsychotics in the general population differs from the suicide group. In the population controls, we observed a slight age gradient.

### Dispensing of antidepressants

Table 7 shows the percentages that were dispensed antidepressants within the last 12 months and within 30 days of suicide. In the last year prior to suicide, 51.2% of females and 36.4% of males were dispensed antidepressants. The equivalent percentages within 30 days of suicide were 21.9% for females and 15.4% for males, respectively. We observed an age gradient in the dispensing of antidepressants for males at both time intervals, while this pattern was less pronounced for females. The percentages with dispensed antidepressants are considerably lower in the general population. In this group, the percentages varied from 9.7% for females and 5.1% for males within the last 12 months, to 2.8% for females and 1.4% for males within the last 30 days.

**Table 7:** Proportion (%) of the suicide population and the general population (control) that were dispensed antidepressants within 12 months and 30 days of suicide by gender and age

	12 months					30 days				
	Control		Suicide			Control		Suicide		
Males	%	n	%	n	z-scores^a^	%	n	%	n	z-scores^a^
15–29	2.3	11,716	21.8	19	**12.13**	0.5	2621	6.9	6	**8.46**
30–44	4.8	25,493	32.3	41	**14.50**	1.2	6570	10.2	13	**9.31**
45–64	6.4	41,001	40.7	61	**17.16**	1.8	11,533	17.3	26	**14.28**
65+	7.5	26,113	52.9	37	**14.42**	2.4	8481	31.4	22	**15.85**
Total	5.1	104,323	36.4	158	**29.64**	1.4	29,205	15.4	67	**24.82**
Females	%	n	%	n	z-scores^a^	%	n	%	n	z-scores^a^
15–29	4.2	19,974	25.8	8	**6.00**	0.9	4663	–	–	**–**
30–44	8.1	41,424	52.4	22	**10.52**	2.1	10,902	21.4	9	**8.72**
45–64	12.1	74,826	61.5	40	**12.21**	3.5	21,678	26.2	17	**9.96**
65+	14.3	62,575	54.5	12	**5.39**	5.0	21,883	22.7	5	**3.81**
Total	9.7	198,799	51.2	82	**17.74**	2.8	59,126	21.9	35	**14.64**

^a^Significant z-scores on a 5% level is presented in bold

## Discussion

In this study we have examined dispensing patterns among all registered Norwegian suicides among men and women aged 15 years and older in 2011. The percentage that were dispensed prescription medication was substantial, both within 12 months and within 30 days of suicide. The percentages varied with sex and age. They were higher for females than for males, and we observed an age gradient. A similar pattern was evident for psychotropic medication. Looking at the different categories of psychotropic medications, dispensing of hypnotics and sedatives, and of antidepressants were slightly more common than dispensing of other categories of psychotropics. As with the dispensing of any medication, the percentages with dispensed psychotropics were higher for females than for males and it increased with age.

The patterns observed in this study align with previous studies reporting high percentages with prescriptions of both any medication and of psychotropic medications [17, 18, 24]. There are however some differences worth noting. For example, we found higher percentages that were dispensed antidepressants and psychotropic medications in general within the last 30 days prior to suicide than reported within the last 3 months in the study by Henriksson and colleagues [17]. Given the similarity of the healthcare systems in Norway and Sweden, it is not likely that this difference reflect unequal access to the healthcare services. Instead, it could reflect different prescription practices between the two countries or differences due to the time that has elapsed between the two studies. Further, the percentages that were dispensed anti-depressant within the 12 last months of suicide in our Norwegian sample were higher than the percentages that were prescribed anti-depressant by GPs in the corresponding time interval in the study by Windfuhr and colleagues [19]. This could reflect different prescription practices between Norway and UK. Further, we observed smaller differences in the dispensing percentages across different categories of psychotropic medications than reported by previous studies [17, 18].

Unlike previous studies, we compared the dispensing patterns of those who died by suicide with population controls at several time intervals prior to suicide. With the exception of the dispensing of antipsychotics, the dispensing percentages in the suicide group followed the same age and sex patterns as in the general population, with higher percentages among females and with increasing age. As expected, the percentages with dispensed prescription medication were higher in the suicide group than in the general population. This difference was particularly striking for the prescription of psychotropics. These large differences were evident across sex and age, and at both the time intervals considered.

Higher percentages with dispensed psychotropic medications in the suicide group is consistent with studies documenting a high prevalence of mental disorders among men and women who die by suicide. It is commonly assumed that 90% of all suicides involve mental disorders of some sort [8]. More recent studies argue that this estimate is too high, and that a considerable share of suicides occur without preceding symptoms of mental disorders [25]. Still, the identification and treatment of mental disorders, including the use of psychotropic medications, remain among the main strategies for preventing suicide [10]. Our study is part of a series of studies demonstrating that healthcare use for mental health problems [6, 7] and the prescription of psychotropic medication [18] prior to suicide are common. From this and similar studies, it is difficult to judge if the percentage with dispensed psychotropic medications meets the potential for prevention. This is in part due to uncertainty about the prevalence of mental disorders in suicide [25], and the extent to which other available and adequate treatments for mental disorders are offered [26]. However, evidence suggests that there is considerable undertreatment of common mental disorders such as depression, anxiety and substance abuse [27, 28]. In Norway, most prescriptions are made by a GP. A recent literature review on suicide prevention in primary care showed that practitioner education and screening for suicide risk are important, albeit insufficient, for effective suicide prevention [29]. The authors called for more collaborative treatment of depression by multidisciplinary teams to reduce the rates of suicidal ideation in primary care patients. To address the potential for prevention more fully, studies examining both healthcare and medication use prior to suicide are highly warranted.

### Strengths and limitations

To our knowledge, this is the first study to address the dispensing of prescribed medications at several time intervals during the last year prior suicide, allowing for comparisons with population-representative controls. Moreover, we use data from a national health registry which enables us to maintain data on the total population, to study small sub-populations with few or no non-responses or other missing data, and with no sample attrition [30].

The main limitation in this study is that our data do not include medications used in hospitals and nursing homes. This leads to an underestimation of the prevalence of actual medication use prior to suicide. Moreover, the dispensing of medication is not equivalent to actual use, we do not have information about medication adherence. Furthermore, the dispensing of psychotropic medications can be considered a proxy for mental health problems in this study. Although the prescriptions are based on a clinical evaluation by a doctor (and hence are an “objective” way of assessing health), medication is only one of several potential treatments for mental illness, and other types of treatments are not considered in this study.

## Conclusion

Our study finds that dispensing of prescription medications, including psychotropic medications, is common prior to suicide. The percentage with dispensed prescription medication increases with age and are higher for females than for males. This pattern was evident both 12 months and 30 days prior to suicide. At both time points, the percentages with dispensed prescription medication were higher in the suicide group than in the general population. This difference was particularly striking for the prescription of psychotropic medications.

The identification and treatment of mental disorders, including the use of psychotropic medications, is considered central in preventing suicide. Our study finds that dispensing of psychotropic medication is common prior to suicide. However, to evaluate to what extent the percentage with dispensed psychotropic medications meets the potential for prevention, future studies should examine both the use of psychotropic medication and the use of other adequate treatments for mental disorders prior to suicide.

## Abbreviation

GP: General practitioner

## References

[1] Organization WH (2014). Preventing Suicide: A global imperative.

[2] Goldsmith SPT, Kleinman AM (2002). Reducing suicide: a national imperative.

[3] Shepard DS, Gurewich D, Lwin AK (2016). Suicide and suicidal attempts in the United States: costs and policy implications. Suicide Life Threat Behav.

[4] Ferrari AJ, Norman RE, Freedman G (2014). The burden attributable to mental and substance use disorders as risk factors for suicide: findings from the global burden of disease study 2010. PLoS One.

[5] Cho J, Lee WJ, Moon KT (2013). Medical care utilization during 1 year prior to death in suicides motivated by physical illnesses. J Prev Med Public Health.

[6] Stene-Larsen K, Reneflot A. Contact with primary and mental health care prior to suicide: A systematic review of the literature from 2000 to 2017. Scandinavian Journal of Public Health. 0(0):1403494817746274. 10.1177/1403494817746274. 10.1177/1403494817746274 29207932

[7] Luoma JB, Martin CE, Pearson JL (2002). Contact with mental health and primary care providers before suicide: a review of the evidence. Am J Psychiatry.

[8] Cavanagh JT, Carson AJ, Sharpe M (2003). Psychological autopsy studies of suicide: a systematic review. Psychol Med.

[9] Hauge LJ, Stene-Larsen K, Grimholt TK (2018). Use of primary health care services prior to suicide in the Norwegian population 2006–2015. BMC Health Serv Res.

[10] Mann J, Apter A, Bertolote J (2005). Suicide prevention strategies: a systematic review. JAMA.

[11] Wohlfarth TD, van Zwieten BJ, Lekkerkerker FJ, et al. Antidepressants use in children and adolescents and the risk of suicide. Eur Neuropsychopharmacol. 16(2):79–83. 10.1016/j.euroneuro.2005.10.004. 10.1016/j.euroneuro.2005.10.004 16298514

[12] Sharma T, Guski LS, Freund N, et al. Suicidality and aggression during antidepressant treatment: systematic review and meta-analyses based on clinical study reports. BMJ. 2016;352. 10.1136/bmj.i65. 10.1136/bmj.i65 PMC4729837 26819231

[13] Kuehn BM (2008). Fda warns of adverse events linked to smoking cessation drug and antiepileptics. JAMA.

[14] Lavigne JE (2003). Suicidal ideation and behavior as adverse events of prescribed medications: an update for pharmacists. J Am Pharm Assoc.

[15] Gorton HC, Webb RT, Kapur N (2016). Non-psychotropic medication and risk of suicide or attempted suicide: a systematic review. BMJ Open.

[16] Lavigne JE, Mathews J, Knox KL (2010). The pharmacology and epidemiology of post-market surveillance for suicide: the case of gabapentin. J Pharm Health Serv Res.

[17] Henriksson S, Boëthius G, Isacsson G (2001). Suicides are seldom prescribed antidepressants: findings from a prospective prescription database in Jämtland county, Sweden, 1985–95. Acta Psychiatr Scand.

[18] Benson T, Corry C, O’Neill S, et al. Use of prescription medication by individuals who died by suicide in Northern Ireland. Archives of Suicide Research. 2017:1–14. 10.1080/13811118.2017.1289870. 10.1080/13811118.2017.1289870 28166453

[19] Windfuhr K, While D, Kapur N (2016). Suicide risk linked with clinical consultation frequency, psychiatric diagnoses and psychotropic medication prescribing in a national study of primary-care patients. Psychol Med.

[20] Pedersen AG, Ellingsen CL (2015). Data quality in the causes of death registry. Tidsskr Nor Laegeforen.

[21] Furu K, Wettermark B, Andersen M (2010). The Nordic countries as a cohort for Pharmacoepidemiological research. Basic Clin Pharmacol Toxicol.

[22] WHO. ICD-10. Psykiske Lidelser og Atferdsforstyrrelser: Gyldendal Akademisk 1999.

[23] Methodology WCCfDS. 2010.

[24] Isacsson G, Boëthius G, Bergman U (1992). Low level of antidepressant prescription for people who later commit suicide: 15 years of experience from a population-based drug database in Sweden. Acta Psychiatr Scand.

[25] Judd F, Jackson H, Komiti A (2012). The profile of suicide: changing or changeable?. Soc Psychiatry Psychiatr Epidemiol.

[26] Layard R (2006). The case for psychological treatment centres. BMJ.

[27] Wang PS, Aguilar-Gaxiola S, Alonso J (2007). Use of mental health services for anxiety, mood, and substance disorders in 17 countries in the WHO world mental health surveys. The Lancet.

[28] Torvik FA, Ystrom E, Gustavson K (2018). Diagnostic and genetic overlap of three common mental disorders in structured interviews and health registries. Acta Psychiatr Scand.

[29] Dueweke AR, Bridges AJ. Suicide interventions in primary care: a selective review of the evidence. Fam Syst Health. 2018. 10.1037/fsh0000349. 10.1037/fsh0000349 29809038

[30] Lyngstad TH, Skardhamar T (2011). Nordic register data and their untapped potential for criminological knowledge. Crime Justice.

